# Time-lapse imaging of identified granule cells in the mouse dentate gyrus after entorhinal lesion *in vitro* reveals heterogeneous cellular responses to denervation

**DOI:** 10.3389/fnana.2024.1513511

**Published:** 2025-01-21

**Authors:** Davide Greco, Alexander Drakew, Nina Rößler, Tassilo Jungenitz, Peter Jedlicka, Thomas Deller

**Affiliations:** ^1^Institute for Clinical Neuroanatomy, Faculty of Medicine, Goethe-University, Frankfurt, Germany; ^2^3R Computer-Based Modelling, Faculty of Medicine, ICAR3R, Justus-Liebig-University, Giessen, Germany

**Keywords:** hippocampal slice cultures, regeneration, perforant path, axotomy, deafferentation, plasticity, time-lapse imaging, heterogeneity

## Abstract

Denervation of neurons is a network consequence of brain injury. The effects of denervation on neurons can be readily studied *in vitro* using organotypic slice cultures of entorhinal cortex and hippocampus. Following transection of the entorhino-dentate projection, granule cells (GCs) are denervated and show on average a transient loss of spines on their denervated distal dendrites but not on their non-denervated proximal dendrites. In the present study, we addressed the question how single GCs and their denervated and non-denervated segments react to entorhinal denervation. Local adeno-associated virus (AAV)-injections were employed to transduce dentate GCs with tdTomato and entorhinal projection neurons with EGFP. This made it possible to visualize both innervating entorhinal fibers and their target neurons and to identify dendritic segments located in the “entorhinal” and the “hippocampal” zone of the dentate gyrus. Confocal time-lapse imaging was used to image distal and proximal segments of single GCs after entorhinal denervation. Time-matched non-denervated cultures served as controls. In line with previous reports, average dendritic spine loss was ~30% (2–4 days post-lesion) in the denervated zone. However, individual GCs showed considerable variability in their response to denervation in both layers, and both decreases as well as increases in spine density were observed at the single cell level. Based on the standard deviations and the effect sizes observed in this study, a computer simulation yielded recommendations for the minimum number of neurons that should be analyzed in future studies using the entorhinal *in vitro* denervation model.

## Introduction

Brain injury causes local damage as well as denervation of neurons in connected brain areas. Denervated neurons respond to their deafferentation with functional and structural adaptations aimed at maintaining homeostasis and network function (Steward, 1989; Deller and Frotscher, 1997). In recent years, we and others have studied these adaptations using organotypic slice cultures of entorhinal cortex and hippocampus (Becker et al., 2012; Bissen et al., 2021; Del Turco et al., 2019; Lenz et al., 2019; Müller et al., 2010; Vlachos et al., 2012a; Vlachos et al., 2012b; Vlachos et al., 2013; Yap et al., 2020). In these complex cultures, the laminar organization of the dentate gyrus is maintained: entorhinal fibers terminate on distal granule cell dendrites in the outer and middle molecular layer (MML), while associative fibers arising from hilar mossy cells terminate on proximal granule cell dendrites in the inner molecular layer (IML) of the dentate gyrus (Del Turco and Deller, 2007; Vlachos et al., 2012a; Del Turco et al., 2019; Hildebrandt-Einfeldt et al., 2021; Paul et al., 2021; Deller et al., 1999; Stanfield et al., 1979). Transection of the entorhino-dentate projection can be reliably performed under visual control, resulting in an extensive and lamina-specific denervation of distal but not proximal granule cell dendrites (Del Turco and Deller, 2007; Vlachos et al., 2012a; Del Turco et al., 2019). This makes it possible to study the response of different dendritic segments of identified single granule cells to the denervation challenge.

Previous work has reported that distal granule cell dendrites respond to *in vitro* entorhinal denervation by losing on average ~30% of their spines (Vlachos et al., 2012a; Bissen et al., 2021). After longer survival times, spine density recovered and returned to baseline levels. In contrast, no spine loss was reported on average for proximal granule cell dendrites (Vlachos et al., 2012a). How single neurons respond to denervation and how spine density changes in the outer and inner molecular layer of single neurons correlate is unknown. However, these data are of interest to understand the variability of granule cell responses to entorhinal denervation *in vitro* and to estimate the number of dendritic segments or granule cells that need to be investigated to obtain meaningful results in studies using this model.

To address these questions, we first used adeno-associated viral tracing to demonstrate the layer-specific innervation of entorhinal fibers in vitro and to estimate the number of granule cell spines innervated by entorhinal axons. Secondly, we labeled single granule cells to visualize and image their proximal and distal dendritic segments over time. Thirdly, we correlated dendritic changes at the single cell level and observed different response types of granule cells to denervation. We conclude that granule cells differ in their response to denervation and that individual granule cells may not show “average” responses. Finally, we performed a computer simulation that demonstrated the interdependency between effect size, standard deviation and sample size.

## Materials and methods

### Animals

Mice used for the preparation of organotypic slice cultures were bred from wildtype mice (WT, C57Bl6/J background) and housed at the animal facility of the Faculty of Medicine, Goethe University, Frankfurt am Main. Animals were maintained on a 12 h light/dark cycle with food and water available ad libitum. The generation of organotypic entorhino-hippocampal tissue cultures from C57Bl6/J mice was performed in accordance with the German animal welfare law and had been declared to the Animal Welfare Officer of Goethe-University, Faculty of Medicine (Wa-2014-35). Every effort was made to minimize the distress and pain of animals.

### Preparation of organotypic slice cultures

Organotypic entorhino-hippocampal slice cultures (OTCs) were prepared from postnatal day 3–4 old WT-mice of either sex as previously described (Del Turco and Deller, 2007). In brief: after rapid decapitation of mice, 300 μm thick horizontal brain slices were generated using a vibratome (LeicaVT1200S). The hippocampi and entorhinal cortices were dissected and placed on a sterile membrane culture insert (Millipore Millicell-CM, PICM ORG, 0.4 μm pore size, 30 mm diameter). Membrane culture inserts with OTCs were placed in 6-well plates containing incubation medium. Culture incubation medium contained 42% MEM, 25% Basal Medium Eagle, 25% head-inactivated normal horse serum, 25 mM HEPES, 0.15% sodium bicarbonate, 0.675% glucose, 0.1 mg/mL streptomycin, 100 U/mL penicillin, 2 mM glutamax. The pH was adjusted to 7.30 and medium was replaced every 2–3 days. Slice cultures were allowed to mature *in vitro* for at least 18 days in a humified incubator (5% CO2 at 35°C). For time-lapse imaging one granule cell was imaged per culture. Cultures were obtained (with one exception per group) from different mouse pups, representing biological replicates.

### Adeno-associated virus (AAV) production

AAV production was performed as described (Yap et al., 2020). In brief, HEK293T cell were transfected with pDP1rs (Plasmid Factory), pDG (Plasmid Factory) and tdTomato or EGFP vector plasmid (12:8:5) by calcium phosphate seeding and precipitation (Grimm et al., 1998). 48 h after transfection, cells were collected, washed with 0.1 M PBS twice, centrifuged at 1500 x g for 5 min and re-suspended in 0.1 M PBS. Subsequently, viral particles were released from the cells by four freeze–thaw cycles. The supernatant was centrifuged at 3200 x g for 10 min to remove the cell debris. The final supernatant was then collected and stored at −80°C.

### Viral labeling

To label dentate granule cells or entorhinal projection cells, organotypic slice cultures were transduced with an AAV serotype 2 (AAV2) containing the gene for tdTomato or EGFP, respectively, under the human synapsin I promotor. Local injections in the dentate gyrus (with tdTomato) or the entorhinal cortex (with EGFP) were performed using an injection pipette pulled from thin-walled borosilicate capillaries (Harvard Apparatus, 30–0066) using a DMZ-Universal Puller (Zeitz-Instrumente). The pipettes were held by microJECT pipette holder with built-in valve (npi electronic) and positioned using a micro-manipulator (Luigs and Neumann). Undiluted solutions of AAV2-hSyn-tdTomato or AAV2-hSyn-EGFP were injected directly into the suprapyramidal blade of the dentate gyrus or into the medial part of the entorhinal cortex (MEC), respectively, by using a pressure application system (npi electronic). For AAV2-hSyn-tdTomato or AAV2-hSyn-EGFP injections, a pressure of 0.04/0.1 bar was applied for 6/8 s, respectively. The dentate gyrus and the MEC were visually identified based on their location and morphology. Slice cultures were visualized with an upright microscope (Nikon FN1) equipped with a camera and software (TrueChrome Metrics) using a 10x water immersion objective lens (Nikon Plan Fluor, NA 0.30). Viral injections were performed 2–3 days after preparation of the slice cultures.

### Transection of entorhinal fibers to the dentate gyrus in OTCs

Entorhinal denervation of organotypic slice cultures was performed as previously described (Del Turco and Deller, 2007). After the initial imaging session on DIV18/19, slice cultures were completely transected from the rhinal fissure to the hippocampal fissure using a sterile scalpel blade. The entorhinal cortex was removed from the culture dish in every denervation experiment in order to ensure complete and permanent separation of the entorhinal cortex. Non-denervated time-matched cultures served as controls.

### Confocal time-lapse imaging

Confocal time-lapse imaging of living granule cells in organotypic slice cultures was performed using an upright confocal microscope (Olympus Fluoview FV3000) with a 60x water immersion objective (Olympus LUMPlanFL N, NA 1.0). The membrane culture insert carrying the slice cultures was placed in a custom-made chamber with imaging buffer consisting of 129 mM NaCl, 4 mM KCl, 1 mM MgCl2, 4.2 mM glucose, 10 mM HEPES buffer solution, 0.1 mM Trolox, 0.1 mg/mL streptomycin, 100 U/mL penicillin, and having a pH of 7.4. Three-dimensional image stacks (0.5 μm step size in the z-axis) of dendritic segments of single granule cells located in the IML or MML of the suprapyramidal layer of the dentate gyrus were acquired using the FV31S-SW software (Olympus). The image stacks were acquired with a 5.5x digital zoom at a resolution of 512×512 pixels (0.075 μm/pixel). Only two dendritic segments per cell/culture were imaged (one IML-segment and one MML-segment) in order to minimize exposure to laser excitation and the related potential phototoxicity. On average, IML-segments were 22 μm in length while MML-segments were 34 μm in length. Dendrites in the IML were distinguished from dendrites in the MML by the absence of EGFP-labeled entorhinal projection fibers. Time-lapse imaging started on DIV18/19 (Day 0). Each segment was imaged again on day 2, 3, 4 and 7. Re-identification was based on nearby landmarks and the shape of neighboring dendrites. For analysis of putative synaptic contacts between EGFP-labeled entorhinal fibers and tdTomato-labeled dendritic granule cell spines, one MML-segment per culture/cell was analyzed on day 0.

### Image processing and data analysis

Image stacks obtained from the confocal microscope were deconvolved using Huygens Professional Version 19.04 (Scientific Volume Imaging). Subsequently, image processing and analysis were performed using Fiji (version 1.53 s, Schindelin et al., 2012) and Microsoft Excel. The analysis was carried out blind with regard to imaging day, position in the molecular layer (IML or MML) and treatment (entorhinal denervation or non-denervated control). One dendritic segment in the IML and one dendritic segment in the MML were analyzed per cell/culture, with a total number of 12 non-denervated control cells/cultures from 11 animals and 9 denervated cells/cultures from 8 animals. Cultures (with one exception in each group) were obtained from different mouse pups. Spine analysis was performed manually based on published criteria (Holtmaat et al., 2009). Image brightness and contrast were adjusted manually to make use of the full range of gray values. Dendritic spines of all shapes emanating laterally in the xy directions and exceeding the dendrite for at least five pixels (0.375 μm) were counted to assess spine density. Spine density is defined as the number of spines on a given dendritic length (#/μm). Spines were counted using an ImageJ Plugin.

### Statistical analysis

In order to analyze the changes in spine density (“Value”) in response to entorhinal lesion (“Treatment”) in dependence of the termination layers (“Layer”) followed up over several days (“Day”) a robust three-way ANOVA for repeated measures was employed. We used the function “RM” of the R-package “MANOVA.RM” (version 0.5.4, Friedrich et al., 2019, R Statistical Software: vR-4.4.1; R Core Team, 2024). Following the recommendations in the discussion of Friedrich et al. (2019), the Wald-type statistic “WTS” was considered, and the *p*-values were obtained by resampling using the wild boot-strap method (“WildBS”). Specifically, the model Value ~ Treatment * Layer * Day with “Layer” and “Day” as intra-subject factors (“Cell”) was analyzed. This was followed by two-way ANOVAs separately for the IML and the MML as Value ~ Treatment * Day. Finally, pairwise post-hoc comparisons of “Treatment” were employed as univariate ANOVAs (function “MANOVA”) on Value—Treatment separately for each Day and each Layer, respectively. *p*-values of these post-hoc comparisons were adjusted using the Benjamini-Hochberg procedure (“BH”) to control the false discovery rate.

Potential within-cell correlations (i) of relative spine density changes of segments located in the IML with segments located in the MML and (ii) of relative spine density changes of the same segments with the day post-lesion (dpl) were assessed using a correlation method designed for repeated measurements (Bakdash and Marusich, 2017). We used the function “rmcorr” of the R-package of the same name (version 0.7.0). This method provides one joint coefficient of correlation (“r”) of repeated paired measurements in the individuals.

Significance level (*α*) was set to *p* = 0.05. If *p* < 0.05, the null hypothesis was rejected. Significant results are marked with an asterisk (*), results that were not significant are marked with “ns” (not significant). Following data processing and analysis, graphical visualization was performed in Graphpad Prism (Version 6.07). Statistical quantities are indicated in the figure legends. Unless stated otherwise, measures of location are given as mean ± standard error of mean (SEM).

Graphical visualization of sample size determination was performed in Matlab (Mathworks, Natrick, MA, version R2023b/R2024). Sample size calculations are based on the following equation (Dell et al., 2002):


$$n=1+2C\;{\left(\frac{s}{d}\right)}^{2}$$

Where s is the standard deviation, d is the effect size and C is a constant based on defined values for *α*- and *β*-errors. We chose a significance level of 5% and a power of 90%. Therefore, *α* = 0.05 and 1-*β* = 0.9. Hence, C = 10.51. The sample size was plotted against different effect size values. In our study, the effect size is the difference of mean spine density changes in the MML relative to initial density on day 0 after ECL compared to time-matched control cultures. The range of the estimated effect size was based on our data and previous publications (Vlachos et al., 2012a; Bissen et al., 2021) demonstrating spine density reduction after ECL *in vitro*. A set of curves were plotted for different standard deviation values.

## Results

### AAV2-hSyn-EGFP injections into the entorhinal cortex and AAV2-hSyn-tdTomato injections into the dentate gyrus label the entorhino-dentate projection and their target granule cells

**Figure 1:**
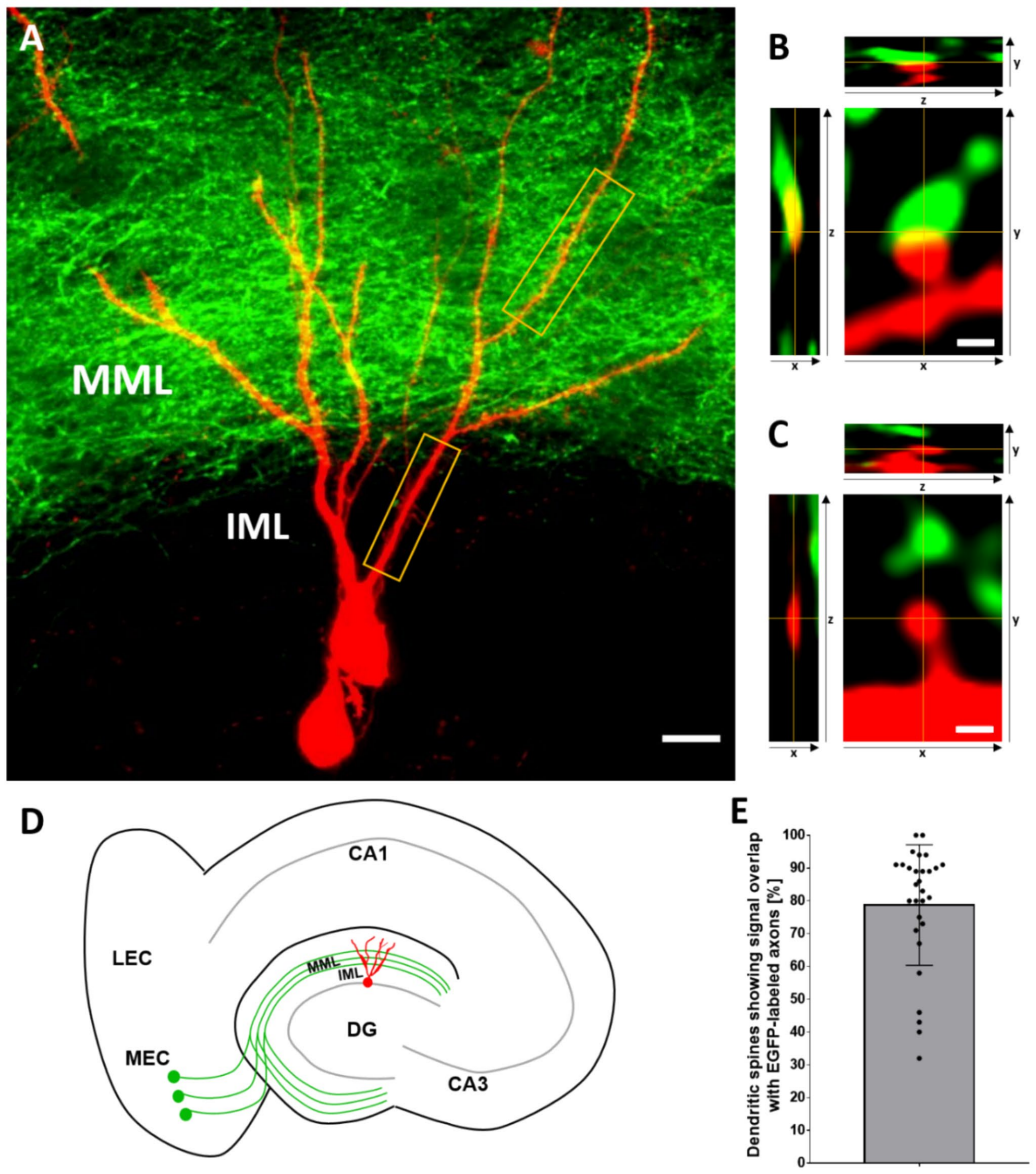
AAV2-hSyn-EGFP injections into the entorhinal cortex and AAV2-hSyn-tdTomato injections into the dentate gyrus label the entorhino-dentate projection and their target granule cells. **(A)** Representative image (maximum intensity projection) of a tdTomato-labeled granule cell (red), receiving EGFP-labeled entorhinal projection fibers (green) in the middle molecular layer (MML). The entorhinal fiber plexus forms a sharp border toward the adjacent inner molecular layer (IML), allowing identification of granule cell dendrites located in the entorhinal and hippocampal zones of the dentate gyrus. Distal and proximal segments belonging to the same cell were selected for time-lapse imaging (yellow boxes). Scale bar: 10 μm. **(B,C)** Representative images of tdTomato-labeled dendritic spines (red) of a granule cell with **(B)** and without **(C)** signal overlap with EGFP-labeled axonal perforant path fibers in all three-dimensional planes. **(D)** Schematic of the entorhino-hippocampal slice culture. In these cultures, the entorhino-dentate projection (green) and the arborization of dentate granule cells (red) are organotypic, i.e., very similar to the in vivo situation. The projection from the medial entorhinal cortex (MEC) to the MML of the dentate gyrus (DG) is illustrated. **(E)** On average, 79% ± 18% (mean ± SD) of granule cell spines on distal dendritic segments of granule cells were contacted by EGFP-positive entorhinal boutons on DIV 18/19 (day 0). *n* = 29 MML-segments in 29 cells from 29 cultures. This analysis also includes segments not used for time-lapse imaging.

Large injections of AAV2-hSyn-EGFP into the medial entorhinal cortex resulted in a dense labeling of the entorhino-dentate projection to the dentate gyrus (Figures 1A,D). In line with the well-described lamination of the dentate gyrus, EGFP-labeled fibers formed a sharp border toward the inner molecular layer, i.e., the termination zone of mossy cells. Local injections with the fluorescent protein tdTomato into the granule cell layer of the dentate gyrus primarily labeled single granule cells (Figures 1A,D). In the case of larger injections, a small number of hilar neurons were also labeled. Granule cells could readily be identified in living cultures on the basis of their location and their dendritic arborization. Proximal dendritic segments located outside the entorhinal fiber plexus could be distinguished from distal segments located within the entorhinal fiber plexus. For the purposes of this study, distal segments located within the densely labeled middle molecular layer were chosen for analysis. These dendritic segments lie within the “entorhinal zone” of the molecular layer of the dentate gyrus. Proximal dendritic segments were selected from the part of the dendritic tree below the entorhinal fiber plexus. This is the zone where mossy cell axons terminate (Blasco-Ibez and Freund, 1997; Scharfman, 2018). These dendritic segments lie within the “hippocampal” zone of the molecular layer of the dentate gyrus.

To estimate the number of entorhinal fibers terminating on granule cell spines, we determined the number of tdTomato-labeled dendritic spines, which are in contact with EGFP-labeled boutons on day 0, i.e., at DIV18/19 (Figures 1B,C). On average 79% of all tdTomato-labeled dendritic spines were contacted by entorhinal boutons (Figure 1E), which is in line with published data on the fraction of spines receiving entorhinal input (Nafstad, 1967; Hjorth-Simonsen and Jeune, 1972; Matthews et al., 1976; Steward and Vinsant, 1983). This finding also suggests that the viral labeling approach we employed here labeled the overwhelming majority of entorhino-dentate projection axons. Of note, in some cases all spines of a dendritic segment were contacted by entorhino-dentate fibers, whereas in other cases only 30–40% of spines of a dendritic segment appeared to receive entorhinal input.

### After entorhinal denervation average spine density of distal dendritic segments decreases whereas average spine density of proximal dendritic segments remains constant

**Figure 2:**
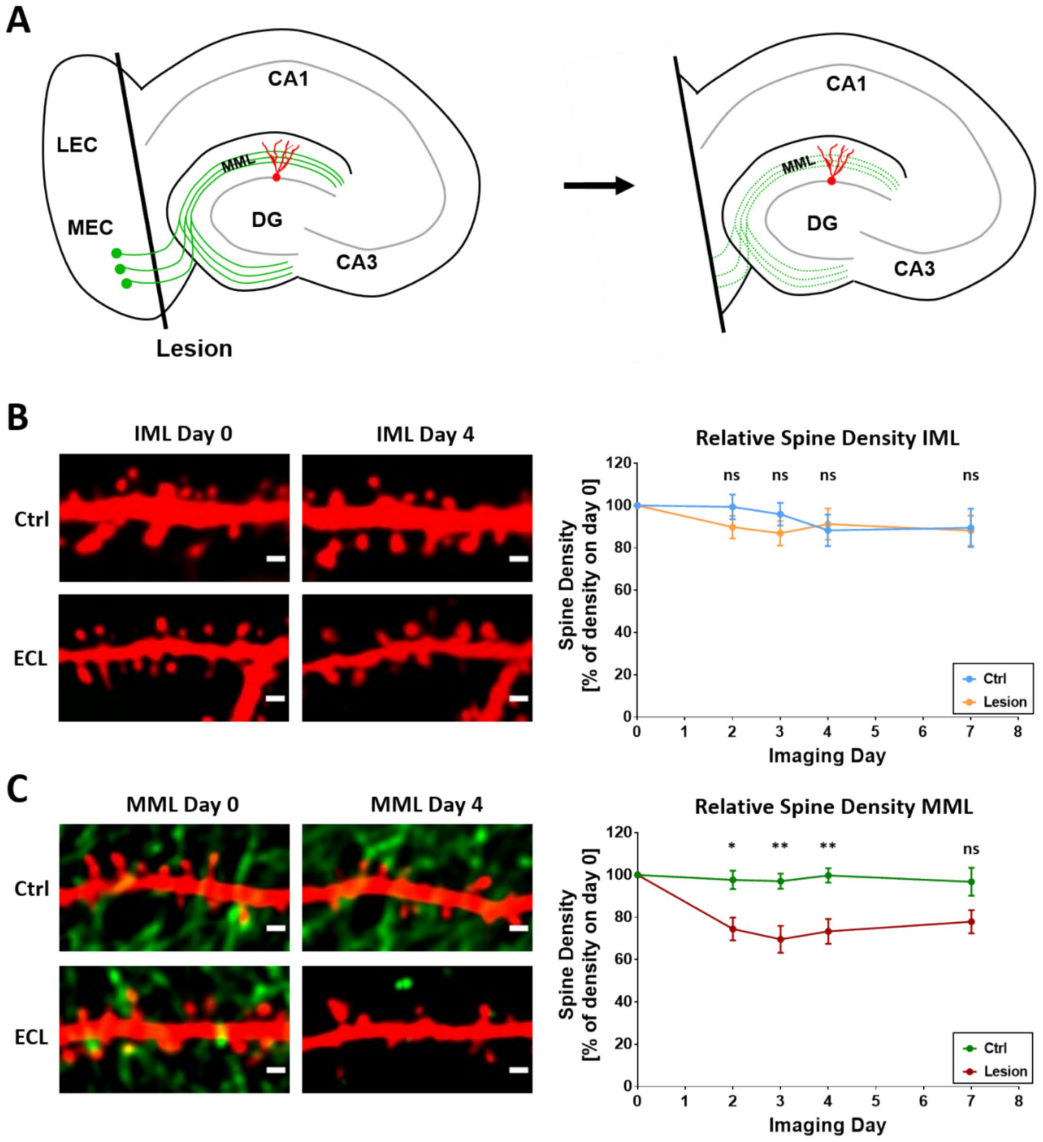
Average spine density of single granule cells decreases in the MML while remaining constant in the IML after entorhinal denervation. **(A)** Schematic of the in vitro entorhinal denervation model. Using a scalpel knife, the entorhinal cortex is cut away from the hippocampus. Following the lesion, entorhinal axons degenerate. Distal dendrites of granule cells located in the middle molecular layer are denervated. Proximal dendrites of granule cells located in the IML are not denervated. **(B,C)** Confocal time-lapse imaging of dendritic segments in the IML **(B)** and MML **(C)**. Imaging started on DIV18/19 (day 0) and was repeated on days 2, 3, 4 and 7. Entorhinal denervation was performed immediately after the first imaging session on day 0. Representative single-plane images (left) show the absence of EGFP-labeled entorhinal fibers in the IML **(B)** and the loss of entorhinal fibers in the MML **(C)** 4 days after lesion. In the IML **(B)** average spine density was not significantly changed (day 2: Ctrl: 100% ± 6% - > Lesion: 90% ± 5%, ns, *p* = 0.35; day 3: 96% ± 5% - > 87% ± 6%, ns, *p* = 0.36; day 4: 88% ± 7% - > 91% ± 7%, ns, *p* = 0.89; day 7: 90% ± 7% - > 88% ± 7%, ns, *p* = 0.91) in both, denervated cultures and non-denervated control cultures. In the MML **(C)**, average spine density significantly decreased after entorhinal denervation compared to non-denervated control cultures (day 2: 98% ± 4% - > 74% ± 5%, *p = 0.014; day 3: 97% ± 4% - > 70% ± 6%, **p = 0.008; day 4: 100% ± 3% - > 73% ± 6%, **p = 0.008; day 7: 97% ± 7% - > 75% ± 6%, ns, *p* = 0.07); post-hoc pairwise comparisons following robust repeated measures ANOVA, *p*-values were adjusted to control FDR; *n* = 12 non-denervated granule cell segments; *n* = 9 denervated granule cell segments. Scale bars: 1 μm.

Transection of the entorhino-dentate projection was performed under visual control and the entorhinal cortex was subsequently removed from the culture dish (Figure 2A). Axotomized entorhinal axons degenerated and lost their fluorescence signal (Figure 2C), confirming the denervation. Proximal as well as distal segments of single granule cells were identified on day 0 and re-identified on days 2, 3, 4, and 7 post-lesion and their spine densities were determined (Figures 2B,C). Control cultures without lesion were imaged in the same way (Figures 2B,C). These cultures served as time-matched controls.

The three-way ANOVA revealed a global effect of entorhinal denervation on spine density (WTS = 5.967, *p* = 0.0146), whereas “Layer” (WTS = 1.752, *p* = 0.1856) and “Day” (WTS = 1.279, *p* = 0.7341) had per se no global effect. However, there was a significant interaction between “Treatment” and “Layer” (WTS = 5.962, *p* = 0.0146), indicating that the effect of the lesion is different for the IML and the MML, and that spine density changes in the MML do not correspond to changes in the IML. Therefore, we next applied two-way ANOVAs separately for the two layers and found no effect of the denervation in the IML (WTS = 0.290, *p* = 0.5905), whereas there was a significant effect of the lesion in the MML (WTS = 15.924, *p* = 0.000066). Again, for both IML and MML, there was no per se effect of “Day” (IML: WTS = 2.140, *p* = 0.5439; MML: WTS = 1.826, *p* = 0.6094) nor a significant interaction of “Treatment” and “Day” (MML: WTS = 1.093, *p* = 0.7789; IML: WTS = 5.083, *p* = 0.1658). This indicates that the effect of the lesion on spine density changes in the MML did not depend on the day post-lesion, even though the reduction of spine density seemed to be more pronounced at days 3 and 4 post-lesion (see below). Next, we compared the effect of the entorhinal lesion on spine density separately for MML and IML and for each day post-lesion. There were no significant differences in the IML (day 2: Mean (Lesion) – mean (Ctrl) = −23.7%, WTS = 1.534, *p* = 0.35; day 3: WTS = 1.273, *p* = 0.36; day 4: WTS = 0.082, *p* = 0.89; day 7: WTS = 0.015, *p* = 0.91) (Figure 2B). In contrast, significant differences in change of spine density post-lesion were obtained in the MML compared to controls at each but day 7 post-lesion (day 2: Mean (Lesion) – mean (Ctrl) = −24%, WTS = 11.233, *p* = 0.0136; day 3: −27%, WTS = 14.243, *p* = 0.0076; day 4: −27%, WTS = 14.987, *p* = 0.0076; day 7: −22%, WTS = 4.927, *p* = 0.0702) (Figure 2B).

### Spine density changes of single granule cell dendritic segments in control and denervated cultures

**Figure 3:**
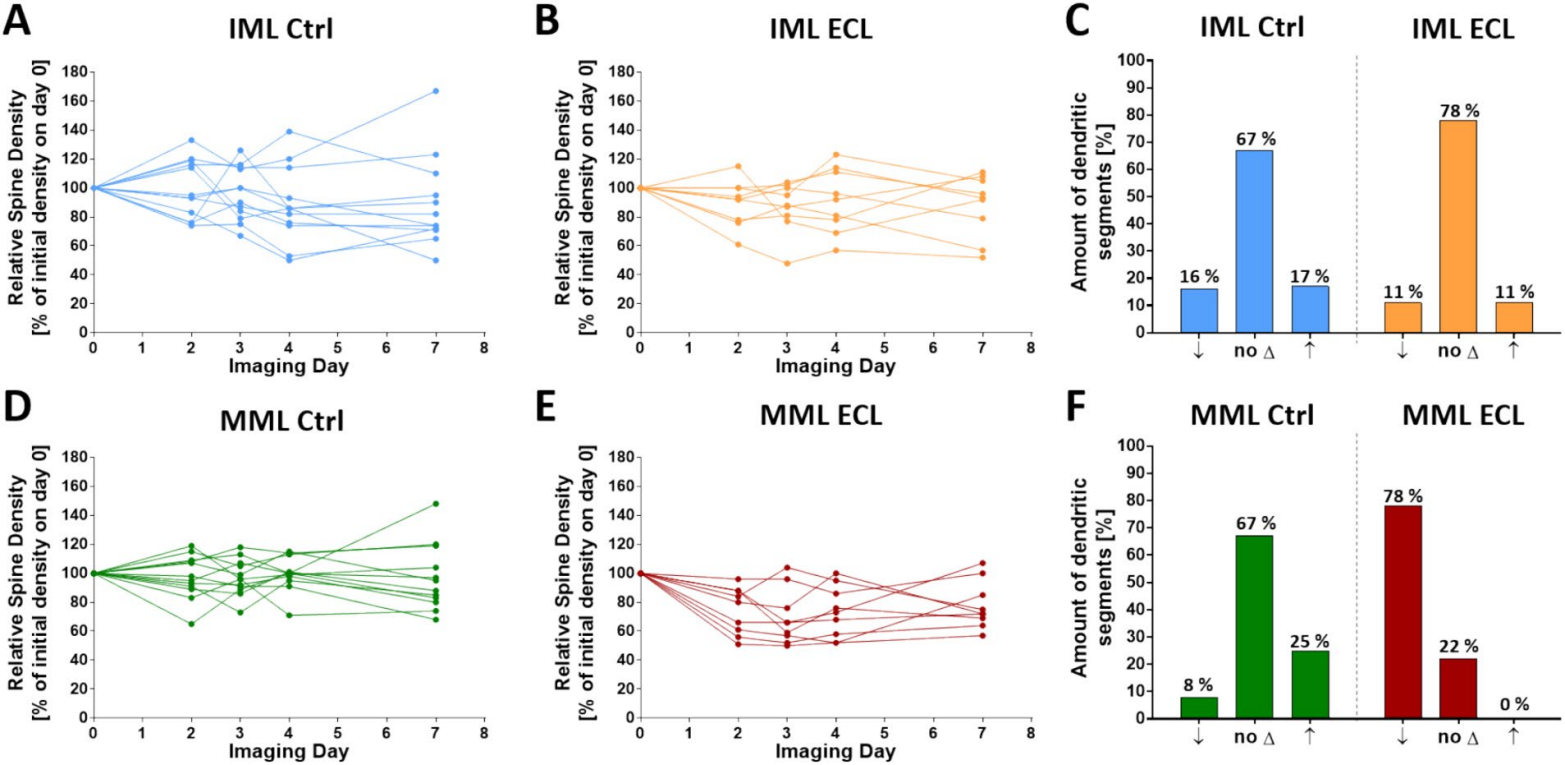
Spine density changes of single granule cell dendritic segments in control and denervated cultures. **(A–C)** In the IML, individual granule cell dendritic segments in control and denervated cultures showed spine density changes during the observation period **(A,B)**. The majority of segments maintained their spine density (no ∆), while some segments lost (↓) or gained (↑) spines at day 4 **(C)**. **(D-F)** In the MML, individual dendritic segments of non-denervated control cultures also showed spontaneous spine density changes **(D)**. The majority of segments maintained their spine density, while some segments lost or gained spines **(F)**. After entorhinal denervation, average spine density in the MML decreased to 73% at 4 dpl, but spine density changes of single segments ranged from 52 to 100% at 4 dpl. In this group, the majority of segments exhibited a loss of spines. Only few segments maintained their spine density and none gained spines at 4 dpl. *n* = 12 non-denervated control cultures and *n* = 9 denervated cultures. Single cell data are shown in Supplementary Figure 1 and Supplementary Table 1.

Analysis of single granule cell dendrites in the middle and the inner molecular layer revealed that spine density changes of single cells did not necessarily reflect average spine density changes. Single segments showed a considerable variety of density changes. This heterogeneity was observed both in the inner molecular layer and middle molecular layer of control as well as lesioned cultures (Figures 3A,B,D,E; single cell data are shown in Supplementary Figure 1 and Supplementary Table 1). Since some spine density fluctuations are to be expected, we defined dendrites within one standard deviation of the time-matched control mean as dendrites without change. Dendritic segments showing an increase of more than one standard deviation were considered to be segments gaining spines, whereas dendritic segments showing a decrease of more than one standard deviation were considered to be segments losing spines. Using these three categories, we found that spine density changes in the inner molecular layer were in an equilibrium in both control as well as cultures 4 days post-lesion (Figure 3C). In the middle molecular layer, the majority of dendrites did not show spine density changes in controls (Figure 3F). In contrast, the majority of these segments showed a reduction in spine density after entorhinal denervation (Figure 3F). Of note, the range of spine density changes was considerable, ranging from 48% loss of spines to no spine loss (Figure 3E).

### Single granule cells show layer-specific spine density changes

**Figure 4:**
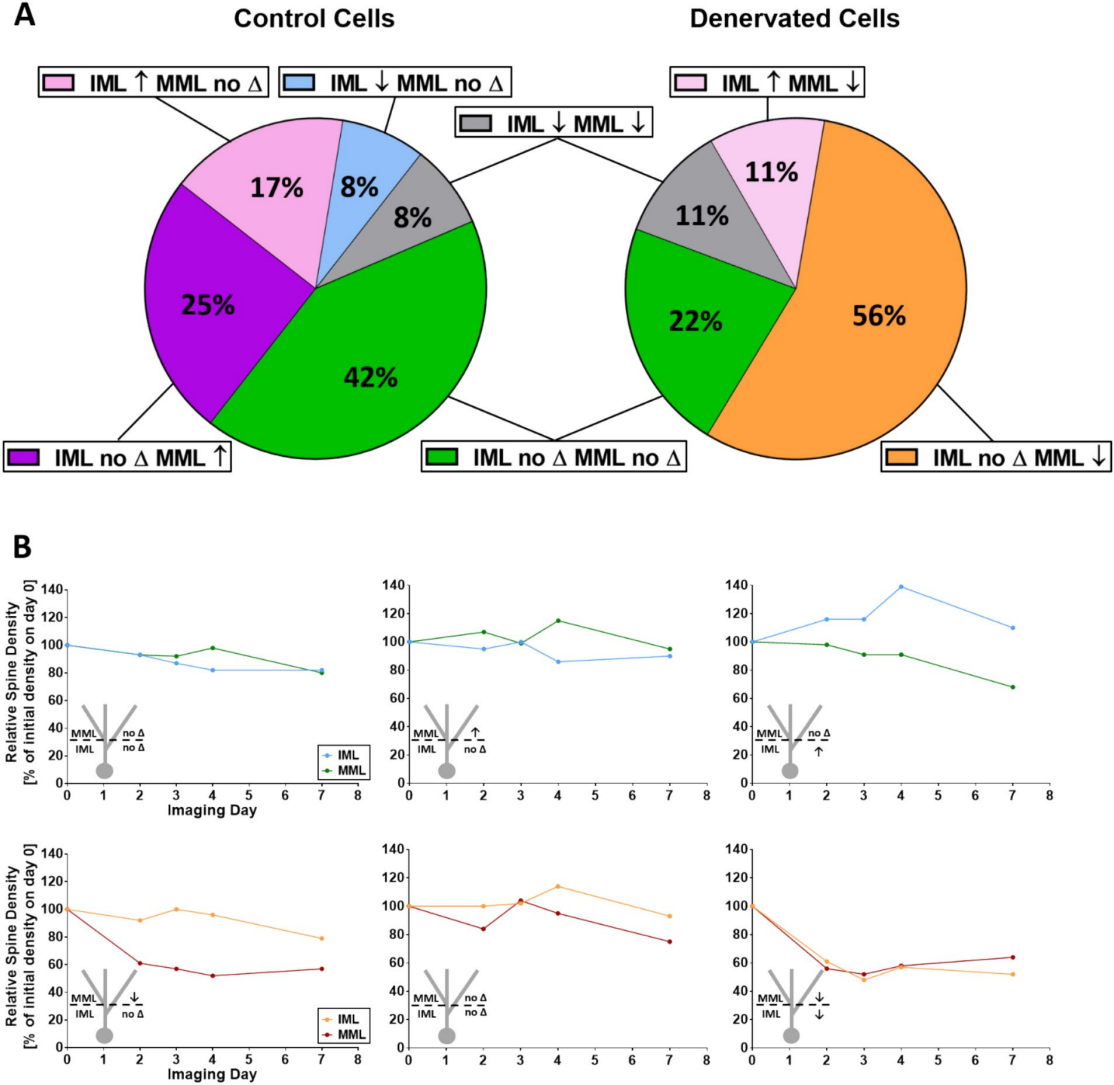
Single granule cells show layer-specific spine density changes. **(A)** Non-denervated control granule cells segments exhibited several combinations of spine density changes in the IML and MML at day 4. Distal and proximal segments lost spines (↓), gained spines (↑) or maintained spine density (no ∆). Spine density changes in the IML were not correlated with changes in the MML (repeated measures correlation; ns). In contrast, denervated cultures showed a different pattern at 4dpl. Of note, although the majority of MML segments lost spines, the IML of such cells showed either no change, a loss of spines or an increase in spines. Spine density changes in both the IML and the MML of single denervated granule cells in both control as well as lesioned cultures were not correlated with the day post-lesion (repeated measures correlation; ns). **(B)** Representative spine density changes in the IML and MML of single granule cells in control (upper row) and denervated (bottom row) cultures for the three most frequent categories. Spine density changes of all single cells are shown in Supplementary Figure 1.

Finally, we related spine density changes of proximal dendritic segments to spine density changes of distal dendritic segments of the same granule cells (Figures 4A,B). Using the same three categories as described in the previous paragraph, we observed five combinations of changes for control segments (Figure 4A). The largest fraction (42%) were stable neurons, which did not show major spine density changes. In contrast, following denervation the largest fraction (56%) belonged to granule cells that lost spines in the middle molecular layer without major changes in the non-denervated inner molecular layer (Figure 4A). These observations raised the question whether spine density changes of distal and proximal dendritic segments of the same cell are correlated. In both control cultures as well as in lesioned cultures, we did not observe a correlation in the IML as well as in the MML, indicating that spine density changes occurring in the two layers are independent of each other irrespective of denervation (repeated measures correlations; control: MML versus IML: r = 0.30, *p* = 0.0708; lesion: MML versus IML: r = 0.24, *p* = 0.2239; control: IML versus dpl: r = −0.26, *p* = 0.1171; lesion: IML versus dpl: r = −0.027, *p* = 0.8918; control: MML versus dpl: r = −0.028, *p* = 0.8695; lesion: MML versus dpl: r = 0.20, *p* = 0.2966).

### Computer simulation revealing the interdependencies of effect sizes (spine loss), standard deviations (segment variability) and sample size

**Figure 5:**
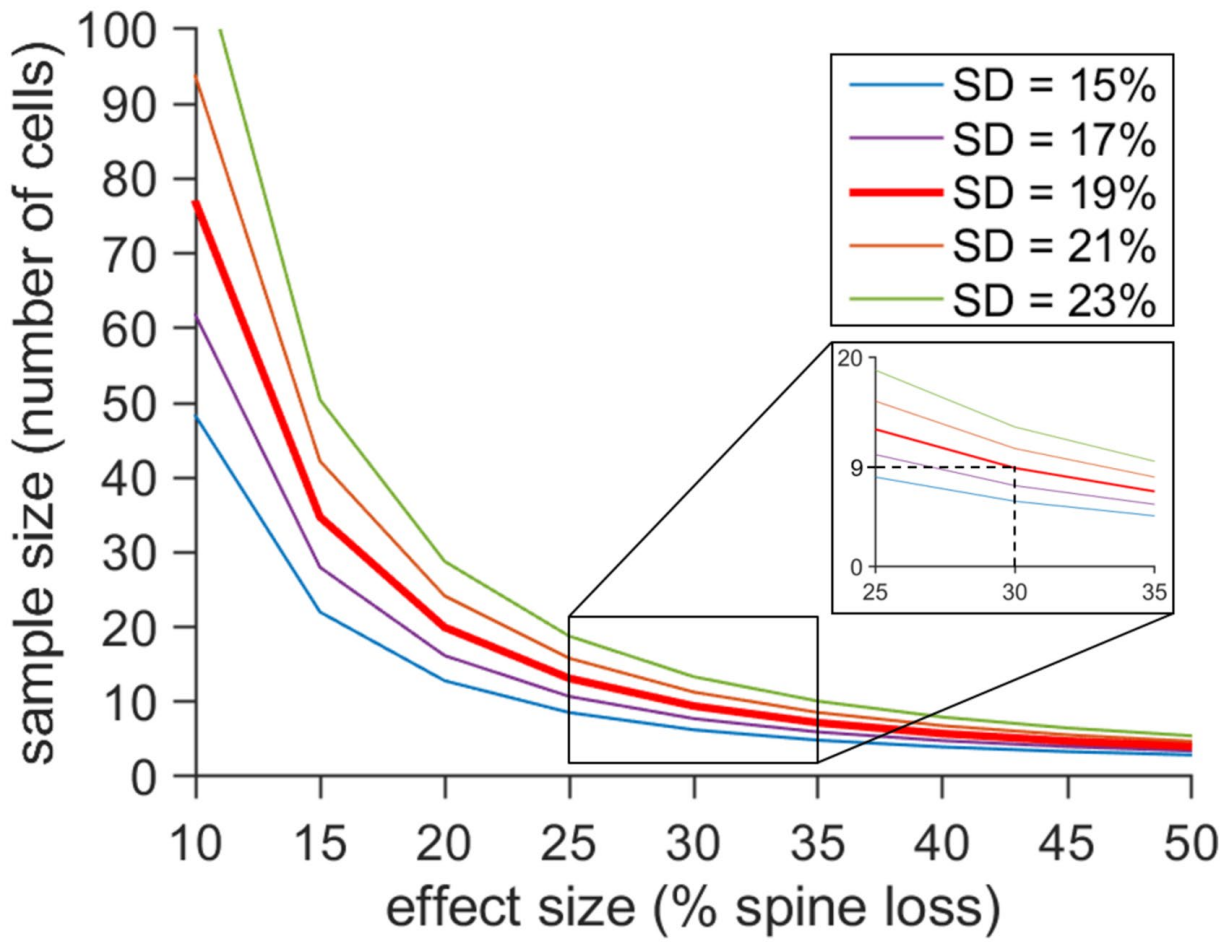
Computer simulation revealing the interdependency of effect size (spine loss), standard deviation (segment variability) and sample size. The predicted sample size (number of granule cells) was calculated for a significance level of 0.05 based on different values for the effect size (% spine loss) and different standard deviations (SD). At 3dpl we observed a maximal spine density reduction of 30% and a SD of 19% in our material. For these parameters a sample size of 9 or more is recommended to achieve significance. Smaller effect sizes and higher SDs increase the number of cells that should be analyzed, while larger effect sizes and smaller SDs have the opposite effect. The calculations shown here will help to design future studies using the in vitro entorhinal denervation model.

Since the response to denervation of dendritic segments in the middle molecular layer varied considerably, we wondered what sample size is required to obtain a significant result (*p* < 0.05) despite this high degree of segment variability. To address this question, we calculated the predicted sample sizes (number of granule cells/dendritic segments) for different effect size values (spine loss) and standard deviations (Figure 5). Matching the results of our simulation with the maximal effect size at 3d post-lesion (−30%; standard deviation 19%), a sample size of *n* = 9 is recommended to obtain a robust result. Thus, the sample sizes used in our study (*n* = 9 denervated cultures, *n* = 12 non-denervated control cultures) appear to be sufficient for analysis.

## Discussion

In the present study, we addressed the question how single granule cells and their denervated and non-denervated segments react to entorhinal denervation. The results of our study can be summarized as follows: (1) AAV-injections into the entorhinal cortex made it possible to distinguish between the entorhinal and hippocampal zones of the molecular layer of the dentate gyrus in living cultures. (2) Approximately 80% of granule cell spines located in the entorhinal zone of the dentate gyrus were in contact with EGFP-positive boutons. (3) Following entorhinal denervation, an average dendritic spine loss of ~30% was observed on granule cell dendrites in the middle molecular layer. On average, no significant spine loss was seen on proximal dendritic segments of granule cells in the non-denervated inner molecular layer. The averaged datasets confirmed data from previous reports using the *in vitro* entorhinal denervation model (Vlachos et al., 2012a; Bissen et al., 2021). (4) Single non-denervated granule cells showed spontaneous spine density fluctuations on their distal and proximal dendrites. (5) Single denervated granule cells showed variable degrees of spine loss on their denervated distal dendrites ranging from no spine loss to very extensive spine loss. The proximal dendrites of these cells showed both increases as well as decreases in their spine density. (6) A computer simulation using the effect sizes and the standard deviation of single granule cell responses to denervation, yielded recommendations for the minimum number of neurons that should be analyzed in future studies using the entorhinal in vitro denervation model.

### EGFP-labeling of entorhino-dentate axons makes it possible to identify granule cell dendrites located in the denervated middle and the non-denervated inner molecular layer of the dentate gyrus

Injections of AAVs containing the genes for the fluorescent proteins tdTomato or EGFP were employed to label dentate granule cells or entorhinal projection neurons, respectively. While sparse labeling of dentate granule cells with tdTomato was aimed to obtain single cells with isolated dendritic segments, EGFP injections into the entorhinal cortex were aimed at labeling as many entorhino-dentate fibers as possible. Indeed, in the cultures used in this study, the EGFP-labeled entorhinal fiber plexus in the middle molecular layer was very dense and clearly delineated the middle molecular layer from the inner molecular layer. In the middle molecular layer, ~79% of granule cell spines formed putative contacts with EGFP-positive entorhinal boutons. This result is in line with previous studies, which reported that ~85% of all granule cell spines form synapses with entorhinal axons (Nafstad, 1967; Hjorth-Simonsen and Jeune, 1972; Matthews et al., 1976; Steward and Vinsant, 1983). Some of the remaining spines may be targeted by fibers from the para- and presubiculum (Köhler, 1985). We conclude that the overwhelming majority of entorhino-dentate fibers targeting the middle molecular layer are EGFP-labeled in our material.

The EGFP-labeled entorhinal plexus made it possible to identify tdTomato-positive granule cell dendrites located in the middle molecular layer prior to the lesion and to follow these dendritic segments over time. Following entorhinal denervation, the EGFP-labeled entorhinal axons degenerated, directly verifying the completeness of the entorhinal lesion in the living cultures. Similarly, proximal granule cell dendrites of the same granule cell located in the inner molecular layer could be reliably identified prior to the lesion. This ensured that only proximal dendritic segments without entorhinal input were imaged and included in our analysis. In sum, EGFP-labeling of the entorhino-dentate projection delineated the layers of the dentate gyrus and helped to distinguish dendritic segments of single granule cells located in the “entorhinal termination zone” or the “hippocampal termination zone” of the molecular layer of the dentate gyrus.

### Non-denervated granule cell dendrites exhibit spontaneous spine density fluctuations *in vitro*

**Figure 6:**
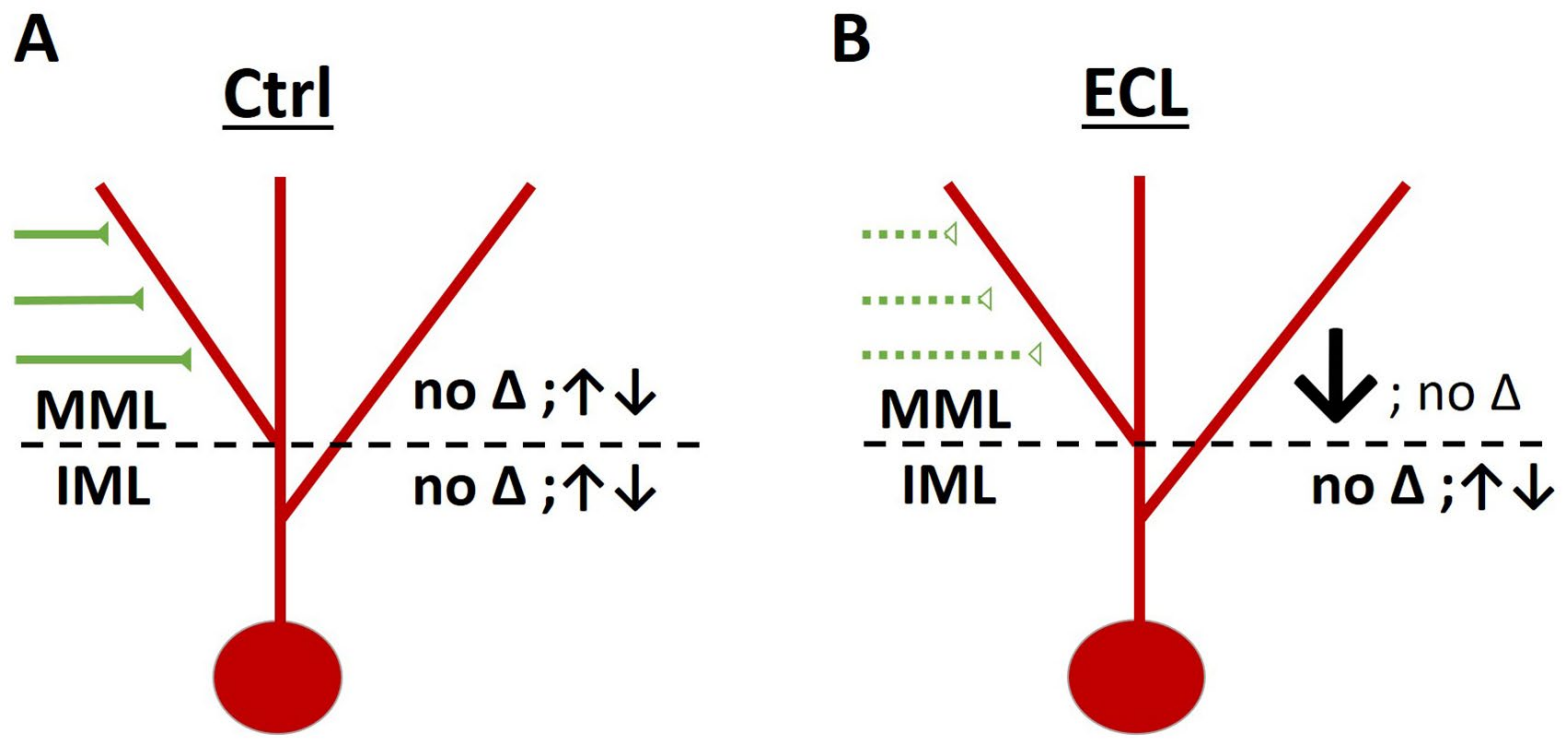
Layer-specific responses of granule cells to entorhinal denervation. **(A)** In control cultures, average spine density was maintained in both the middle molecular layer (MML) as well as in the inner molecular layer (IML) throughout the observation period. However, spine density of individual dendritic segments varied, and some segments lost or gained spines. Spine density changes in the MML and IML were not correlated. **(B)** Following entorhinal denervation, dendritic segments in the MML were denervated. In this layer the majority of segments lost spines and the average spine density decreased (~30% day 3 and 4 post-lesion). In contrast, dendritic segments in the non-denervated IML exhibited spine density fluctuations similar to control cultures. Spine density changes in the MML and IML were not correlated.

Previous studies using organotypic hippocampal slice cultures have reported that these cultures continue to mature *in vitro*, similar to the *in vivo* situation (Li et al., 1993; Zimmer and Gähwiler, 1984; Gähwiler, 1984; Caeser and Aertsen, 1991; Paul et al., 2021; Radic et al., 2017). By 3 weeks in vitro, i.e., the starting time point for the lesion experiments in our study, the cultures exhibit a young adult morphology. In these cultures, the laminar organization of the dentate gyrus and hippocampus is present and granule cell spines, which are highly dynamic and immature during the first 2–3 weeks in vitro have stabilized and acquired mature shapes (Dailey and Smith, 1996; Ziv and Smith, 1996). Nevertheless, even at this age, the cultures are dynamic and imaging of granule cell dendrites in the entorhinal and in the hippocampal zones of the dentate gyrus revealed spontaneous spine density fluctuations in both layers. Of note, spine density increases as well as decreases were seen in both layers and these changes were not correlated, i.e., spine density changes in the two zones occurred independent of each other (Figure 6).

Although organotypic cultures reach a young adult morphology by 3 weeks *in vitro*, they continue to mature (Paul et al., 2021; Radic et al., 2017). To take these maturation effects into account, we have used time-matched non-denervated cultures as controls for the lesioned cultures. This strategy, which we and others have employed previously (e.g., Vlachos et al., 2012a; Bissen et al., 2021), appears to be important to avoid maturation-dependent bias which may occur if cultures of different maturation age are compared. In addition, we have defined an increase or a decrease in spine density as a change in spine density that is greater than the mean spine density of control segments +/− one standard deviation. In this way, both the continuing maturation of the cultures as well as background changes of spine densities were taken into account in our analysis.

### Heterogeneity of the granule cell response to denervation

Following denervation, granule cell dendrites located in the middle molecular layer showed an average loss of ~30% of their spines (27%; 4 days post-lesion). This confirms previous *in vivo* (Vuksic et al., 2011) as well as in vitro studies (Vlachos et al., 2012a; Bissen et al., 2021), which reported an average loss of ~35% of granule cell spines for the same time point. Of note, the average loss of spines is less than the average loss of entorhinal synapses, which has also been reported following entorhinal denervation in vivo for both rats and mice (Parnavelas et al., 1974; Caceres and Steward, 1983; Vuksic et al., 2011). This mismatch may be the result of several homeostatic mechanisms counteracting the loss of synapses and spines. For example, new spines are constantly formed following denervation (Vlachos et al., 2012a), sprouting and reactive synaptogenesis (Prang et al., 2003; Paul et al., 2021) may reinnervate denervated spines, and large mushroom-like spines are more stable than others (Caceres and Steward, 1983; Yap et al., 2020). Thus, the spine loss observed at a given time point does not equal the loss of synapses, it rather reflects the net sum of several mechanisms resulting in an average effect size of ~30% spine loss in the *in vitro* model.

Compared to the average spine loss, individual granule cell segments exhibited a wide range of spine density changes ranging from ~50% spine loss to no loss of spines. At present, the heterogeneity of dendritic responses to denervation is poorly understood and both pre- as well as postsynaptic explanations may underlie this phenomenon. Regarding the presynaptic side, our tracing data (this study) and previous reports (Nafstad, 1967; Hjorth-Simonsen and Jeune, 1972; Matthews et al., 1976; Steward and Vinsant, 1983) suggest that on average ~ 80–85% of granule cell spines are contacted by entorhinal axons. However, if single dendritic segments are considered, some show 100% of their spines in contact with entorhinal boutons, whereas in other cases only 30–40% of spines of a segment are contacted by entorhinal fibers (Figure 1E). Thus, following entorhinal denervation some dendritic segments may be more extensively denervated than others, resulting in a more pronounced loss of spines. This difference in entorhinal innervation between individual granule cells may be linked to their maturity. Granule cells continue to be born postnatally, and these granule cells mature over time. Postnatally born granule cells form their first spines at 10–12 days of age (Zhao et al., 2006) and receive their entorhinal input later, i.e., around 21 days of age *in vivo* (Vivar and van Praag, 2013; Deshpande et al., 2013; Bergami et al., 2015). As we have shown in a previous publication (Radic et al., 2017), immature granule cells are still present in the slice cultures around 3 weeks *in vitro* and these cells may be less innervated by entorhinal axons. Accordingly, such cells may show a much weaker response to denervation compared to fully mature granule cells.

Regarding the postsynaptic side, several additional possibilities exist: First, some denervated granule cells may be more active than others (Pernía-Andrade and Jonas, 2014), because they are activated by the remaining network, e.g., hippocampal mossy cell axons terminating on proximal granule cell dendrites (Blasco-Ibez and Freund, 1997; Scharfman, 2018). This may result in backpropagating action potentials, which could help to maintain spines in the absence of afferent input. Second, dendritic segments may act as computational units (Govindarajan et al., 2011; Branco and Häusser, 2010), which may differ not only with regard to their plasticity (Govindarajan et al., 2006; Takahashi et al., 2012), but also in their response to denervation. Third, some dendritic spines are more stable than others, because they contain stabilizing proteins, such as the actin-modulating protein synaptopodin (Mundel et al., 1997; Deller et al., 2000). Spines containing synaptopodin persist longer after in vitro entorhinal denervation (Yap et al., 2020) and some segments may have more synaptopodin-positive spines than others. Future studies focusing on these aspects will shed light on these intriguing possibilities, which are not mutually exclusive.

Finally, we also analyzed the effect of entorhinal denervation on proximal granule cell dendrites located in the non-denervated IML. On average, this layer did not show a significant spine loss compared to age-matched control cultures. This is in line with the extant *in vivo* (Vuksic et al., 2011) and in vitro literature (e.g., Vlachos et al., 2012a; Bissen et al., 2021). Similarly, at the level of single cells, we did not find that entorhinal denervation in vitro caused concomitant spine density changes on proximal dendrites in the inner molecular layer (Figure 6). Although these data suggest that the inner molecular layer is unaffected by denervation, evidence has been provided that synaptic remodeling occurs at the ultrastructural level (Hoff et al., 1981; Deller et al., 2006; Kruse et al., 2024). Using the same organotypic culture system and electron microscopy, Kruse et al. (2024) reported that the volume of spines on proximal dendrites was decreased and that these spines form more postsynaptic densities than non-denervated controls. In sum, we conclude that proximal dendrites of denervated granule cells maintain their spines at normal densities but remodel their axo-spinous synapses at the ultrastructural level (Kruse et al., 2024) to adapt to the denervation challenge.

### The heterogeneity of granule cell responses—consequences for future studies

The heterogeneity of granule cell responses to denervation reported in this study may not be quite as large in vivo. Compared to the brain of adult rats and mice, entorhino-hippocampal slice cultures are younger, contain immature granule cells (Radic et al., 2017) and are highly plastic (Del Turco et al., 2019). Although the complex cultures used in our study are organotypic with regard to the entorhino-dentate projection and the arborization of granule cells (Zafirov et al., 1994; Frotscher et al., 1995; Drakew et al., 1999), they are brain isolates and may exhibit some aberrant connectivities. Slice culture systems are useful to study principles of reorganization, but cannot reveal the ground truth. *In vivo* time-lapse imaging of denervated granule cells is needed to reveal the heterogeneity of granule cell responses in living animals.

The heterogeneity of granule cell responses to denervation has practical consequences for the design of future studies using this model. As demonstrated in our simulation (Figure 5), the number of cells needed to detect spine density differences between the control and the lesioned group depends on the effect size as well as the standard deviation of the sample. The smaller the effect size and the larger the standard deviation, the more cells are required. Based on the effect sizes and standard deviations observed in this study, analysis of 10 or more granule cells in control and lesioned cultures each appear to be a reasonable recommendation for the sample size of future studies on spine density changes after denervation.

Furthermore, the heterogeneity of responses of individual dendritic segments suggests that sampling of single dendritic segments may not suffice to capture the response of a granule cell to a given treatment, in our case entorhinal denervation. Sampling of more than one dendritic segment per layer per cell may be a promising strategy. For this, however, optimized imaging sequences and rapid dendritic re-identification strategies need to be established to perform repeated multisampling of dendritic segments at a resolution sufficient for spine analysis without causing phototoxic damage to the imaged neurons.

The heterogeneity of structural and functional properties in dentate granule cells has recently been shown to promote their physiological performance and robustness (Schneider et al., 2023; Kumari and Narayanan, 2024). In addition, recent work has demonstrated that heterogeneity of neuronal responses underlies protection against pathological network changes (Rich et al., 2022; Gast et al., 2024). Future work is needed to determine whether the heterogeneity of granule cell responses to denervation also increases the resilience and robustness of the dentate gyrus network.

## Data Availability

The original contributions presented in the study are included in the article/Supplementary material, further inquiries can be directed to the corresponding author.

## References

[ref1] Bakdash J. Z. Marusich L. R. (2017). Repeated measures correlation. Front. Psychol. 8:456. doi: 10.3389/fpsyg.2017.00456 28439244 PMC5383908

[ref2] Becker D. Willems L. M. Vnencak M. Zahn N. Schuldt G. Jedlicka P.. (2012). Functional and structural properties of dentate granule cells with hilar basal dendrites in mouse entorhino-hippocampal slice cultures. PLoS One 7:e48500. doi: 10.1371/journal.pone.0048500, PMID: 23144894 PMC3492458

[ref3] Bergami M. Masserdotti G. Temprana S. G. Motori E. Eriksson T. M. Göbel J.. (2015). A critical period for experience-dependent remodeling of adult-born neuron connectivity. Neuron 85, 710–717. doi: 10.1016/j.neuron.2015.01.001, PMID: 25661179

[ref4] Bissen D. Kracht M. K. Foss F. Hofmann J. Acker-Palmer A. (2021). EphrinB2 and GRIP1 stabilize mushroom spines during denervation-induced homeostatic plasticity. Cell Rep. 34:108923. doi: 10.1016/j.celrep.2021.108923, PMID: 33789115 PMC8028307

[ref5] Blasco-Ibez J. M. Freund T. F. (1997). Distribution, ultrastructure, and connectivity of calretinin-immunoreactive mossy cells of the mouse dentate gyrus. Hippocampus 7, 307–320. doi: 10.1002/(SICI)1098-1063(1997)7:3<307::AID-HIPO6>3.0.CO;2-H, PMID: 9228528

[ref6] Branco T. Häusser M. (2010). The single dendritic branch as a fundamental functional unit in the nervous system. Curr. Opin. Neurobiol. 20, 494–502. doi: 10.1016/j.conb.2010.07.009, PMID: 20800473

[ref7] Caceres A. Steward O. (1983). Dendritic reorganization in the denervated dentate gyrus of the rat following entorhinal cortical lesions: a Golgi and electron microscopic analysis. J. Comp. Neurol. 214, 387–403. doi: 10.1002/cne.902140404

[ref8] Caeser M. Aertsen A. (1991). Morphological organization of rat hippocampal slice cultures. J. Comp. Neurol. 307, 87–106. doi: 10.1002/cne.903070109, PMID: 1713228

[ref9] Dailey M. E. Smith S. J. (1996). The dynamics of dendritic structure in developing hippocampal slices. J. Neurosci. 16, 2983–2994. doi: 10.1523/JNEUROSCI.16-09-02983.1996, PMID: 8622128 PMC6579052

[ref10] Del Turco D. Deller T. (2007). Organotypic entorhino-hippocampal slice cultures--a tool to study the molecular and cellular regulation of axonal regeneration and collateral sprouting in vitro. Methods Mol. Biol. 399, 55–66. doi: 10.1007/978-1-59745-504-6_5, PMID: 18309925

[ref11] Del Turco D. Paul M. H. Beeg Moreno V. J. Hildebrandt-Einfeldt L. Deller T. (2019). Re-innervation of the Denervated dentate gyrus by sprouting associational and commissural mossy cell axons in Organotypic tissue cultures of entorhinal cortex and Hippocampus. Front. Mol. Neurosci. 12:270. doi: 10.3389/fnmol.2019.00270, PMID: 31798410 PMC6861856

[ref12] Dell R. B. Holleran S. Ramakrishnan R. (2002). Sample size determination. ILAR J 43, 207–213. doi: 10.1093/ilar.43.4.207, PMID: 12391396 PMC3275906

[ref13] Deller T. Bas Orth C. Vlachos A. Merten T. Del Turco D. Dehn D.. (2006). Plasticity of synaptopodin and the spine apparatus organelle in the rat fascia dentata following entorhinal cortex lesion. J. Comp. Neurol. 499, 471–484. doi: 10.1002/cne.21103, PMID: 16998909

[ref14] Deller T. Drakew A. Frotscher M. (1999). Different primary target cells are important for fiber lamination in the fascia dentata: a lesson from reeler mutant mice. Exp. Neurol. 156, 239–253. doi: 10.1006/exnr.1999.7020, PMID: 10328933

[ref15] Deller T. Frotscher M. (1997). Lesion-induced plasticity of central neurons: sprouting of single fibres in the rat hippocampus after unilateral entorhinal cortex lesion. Prog. Neurobiol. 53, 687–727. doi: 10.1016/S0301-0082(97)00044-0, PMID: 9447617

[ref16] Deller T. Mundel P. Frotscher M. (2000). Potential role of synaptopodin in spine motility by coupling actin to the spine apparatus. Hippocampus 10, 569–581. doi: 10.1002/1098-1063(2000)10:5<569::AID-HIPO7>3.0.CO;2-M, PMID: 11075827

[ref17] Deshpande A. Bergami M. Ghanem A. Conzelmann K.-K. Lepier A. Götz M.. (2013). Retrograde monosynaptic tracing reveals the temporal evolution of inputs onto new neurons in the adult dentate gyrus and olfactory bulb. Proc. Natl. Acad. Sci. USA 110, E1152–E1161. doi: 10.1073/pnas.1218991110, PMID: 23487772 PMC3607028

[ref18] Drakew A. Frotscher M. Heimrich B. (1999). Blockade of neuronal activity alters spine maturation of dentate granule cells but not their dendritic arborization. Neuroscience 94, 767–774. doi: 10.1016/S0306-4522(99)00378-4, PMID: 10579567

[ref19] Friedrich S. Konietschke F. Pauly M. (2019). Resampling-based analysis of multivariate data and repeated measures designs with the R package MANOVA. RM 11:051. doi: 10.32614/RJ-2019-051

[ref20] Frotscher M. Zafirov S. Heimrich B. (1995). Development of identified neuronal types and of specific synaptic connections in slice cultures of rat hippocampus. Prog. Neurobiol. 45, 143–164. doi: 10.1016/0301-0082(94)00040-O, PMID: 7598766

[ref21] Gähwiler B. H. (1984). Development of the hippocampus in vitro: cell types, synapses and receptors. Neuroscience 11, 751–760. doi: 10.1016/0306-4522(84)90192-1, PMID: 6330608

[ref22] Gast R. Solla S. A. Kennedy A. (2024). Neural heterogeneity controls computations in spiking neural networks. Proc. Natl. Acad. Sci. 121:8851121. doi: 10.1073/pnas.2311885121, PMID: 38198531 PMC10801870

[ref23] Govindarajan A. Israely I. Huang S.-Y. Tonegawa S. (2011). The dendritic branch is the preferred integrative unit for protein synthesis-dependent LTP. Neuron 69, 132–146. doi: 10.1016/j.neuron.2010.12.008, PMID: 21220104 PMC3032443

[ref24] Govindarajan A. Kelleher R. J. Tonegawa S. (2006). A clustered plasticity model of long-term memory engrams. Nat. Rev. Neurosci. 7, 575–583. doi: 10.1038/nrn1937, PMID: 16791146

[ref25] Grimm D. Kern A. Rittner K. Kleinschmidt J. A. (1998). Novel tools for production and purification of recombinant Adenoassociated virus vectors. Hum. Gene Ther. 9, 2745–2760. doi: 10.1089/hum.1998.9.18-2745, PMID: 9874273

[ref26] Hildebrandt-Einfeldt L. Yap K. Paul M. H. Stoffer C. Zahn N. Drakew A.. (2021). Crossed Entorhino-dentate projections form and terminate with correct layer-specificity in Organotypic slice cultures of the mouse Hippocampus. Front. Neuroanat. 15:637036. doi: 10.3389/fnana.2021.637036, PMID: 33643001 PMC7904698

[ref27] Hjorth-Simonsen A. Jeune B. (1972). Origin and termination of the hippocampal perforant path in the rat studied by silver impregnation. J. Comp. Neurol. 144, 215–231. doi: 10.1002/cne.901440206, PMID: 4112908

[ref28] Hoff S. F. Scheff S. W. Kwan A. Y. Cotman C. W. (1981). A new type of lesion-induced synaptogenesis: I. Synaptic turnover in non-denervated zones of the dentate gyrus in young adult rats. Brain Res. 222, 1–13. doi: 10.1016/0006-8993(81)90936-7, PMID: 7296257

[ref29] Holtmaat A. Bonhoeffer T. Chow D. K. Chuckowree J. Hofer S. B. Hübener M.. (2009). Long-term, high-resolution imaging in the mouse neocortex through a chronic cranial window. Nat. Protoc. 4, 1128–1144. doi: 10.1038/nprot.2009.89, PMID: 19617885 PMC3072839

[ref30] Köhler C. (1985). Intrinsic projections of the retrohippocampal region in the rat brain. I. The subicular complex. J. Comp. Neurol. 236, 504–522. doi: 10.1002/cne.902360407, PMID: 3902916

[ref31] Kruse P. Brandes G. Hemeling H. Huang Z. Wrede C. Hegermann J.. (2024). Synaptopodin regulates denervation-induced plasticity at hippocampal mossy Fiber synapses. Cells 13:114. doi: 10.3390/cells13020114, PMID: 38247806 PMC10814840

[ref32] Kumari S. Narayanan R. (2024). Ion-channel degeneracy and heterogeneities in the emergence of signature physiological characteristics of dentate gyrus granule cells. J. Neurophysiol. 132, 991–1013. doi: 10.1152/jn.00071.2024, PMID: 39110941

[ref33] Lenz M. Galanis C. Kleidonas D. Fellenz M. Deller T. Vlachos A. (2019). Denervated mouse dentate granule cells adjust their excitatory but not inhibitory synapses following in vitro entorhinal cortex lesion. Exp. Neurol. 312, 1–9. doi: 10.1016/j.expneurol.2018.10.013, PMID: 30401642

[ref34] Li D. Field P. M. Starega U. Li Y. Raisman G. (1993). Entorhinal axons project to dentate gyrus in organotypic slice co-culture. Neuroscience 52, 799–813. doi: 10.1016/0306-4522(93)90530-S, PMID: 7680800

[ref35] Matthews D. A. Cotman C. Lynch G. (1976). An electron microscopic study of lesion-induced synaptogenesis in the dentate gyrus of the adult rat. I. Magnitude and time course of degeneration. Brain Res. 115, 1–21. doi: 10.1016/0006-8993(76)90819-2, PMID: 974734

[ref36] Müller C. M. Vlachos A. Deller T. (2010). Calcium homeostasis of acutely denervated and lesioned dentate gyrus in organotypic entorhino-hippocampal co-cultures. Cell Calcium 47, 242–252. doi: 10.1016/j.ceca.2009.12.006, PMID: 20053446

[ref37] Mundel P. Heid H. W. Mundel T. M. Krüger M. Reiser J. Kriz W. (1997). Synaptopodin: an actin-associated protein in telencephalic dendrites and renal podocytes. J. Cell Biol. 139, 193–204. doi: 10.1083/jcb.139.1.193, PMID: 9314539 PMC2139823

[ref38] Nafstad P. H. (1967). An electron microscope study on the termination of the perforant path fibres in the hippocampus and the fascia dentata. Zeitschrift fur Zellforschung Mikroskopische Anatomie 76, 532–542. 10.1007/BF00339754 5585500

[ref39] Parnavelas J. G. Lynch G. Brecha N. Cotman C. W. Globus A. (1974). Spine loss and regrowth in hippocampus following deafferentation. Nature 248, 71–73. doi: 10.1038/248071a0, PMID: 4818565

[ref40] Paul M. H. Hildebrandt-Einfeldt L. Beeg Moreno V. J. Del Turco D. Deller T. (2021). Maturation-dependent differences in the re-innervation of the Denervated dentate gyrus by sprouting associational and commissural mossy cell axons in Organotypic tissue cultures of entorhinal cortex and Hippocampus. Front. Neuroanat. 15:682383. doi: 10.3389/fnana.2021.682383, PMID: 34122019 PMC8194403

[ref41] Pernía-Andrade A. J. Jonas P. (2014). Theta-gamma-modulated synaptic currents in hippocampal granule cells in vivo define a mechanism for network oscillations. Neuron 81, 140–152. doi: 10.1016/j.neuron.2013.09.046, PMID: 24333053 PMC3909463

[ref42] Prang P. Del Turco D. Deller T. (2003). Associational sprouting in the mouse fascia dentata after entorhinal lesion in vitro. Brain Res. 978, 205–212. doi: 10.1016/S0006-8993(03)02836-1, PMID: 12834915

[ref43] R Core Team (2024). _R: A language and environment for statistical Computing_. Vienna: R Foundation for Statistical Computing.

[ref44] Radic T. Jungenitz T. Singer M. Beining M. Cuntz H. Vlachos A.. (2017). Time-lapse imaging reveals highly dynamic structural maturation of postnatally born dentate granule cells in organotypic entorhino-hippocampal slice cultures. Sci. Rep. 7:43724. doi: 10.1038/srep43724, PMID: 28256620 PMC5335612

[ref45] Rich S. Moradi Chameh H. Lefebvre J. Valiante T. A. (2022). Loss of neuronal heterogeneity in epileptogenic human tissue impairs network resilience to sudden changes in synchrony. Cell Rep. 39:110863. doi: 10.1016/j.celrep.2022.110863, PMID: 35613586

[ref46] Scharfman H. E. (2018). Advances in understanding hilar mossy cells of the dentate gyrus. Z. Zellforsch. Mikrosk. Anat. 373, 643–652. doi: 10.1007/s00441-017-2750-5, PMID: 29222692 PMC5993616

[ref47] Schindelin J. Arganda-Carreras I. Frise E. Kaynig V. Longair M. Pietzsch T.. (2012). Fiji: an open-source platform for biological-image analysis. Nat. Methods 9, 676–682. doi: 10.1038/nmeth.2019, PMID: 22743772 PMC3855844

[ref48] Schneider M. Bird A. D. Gidon A. Triesch J. Jedlicka P. Cuntz H. (2023). Biological complexity facilitates tuning of the neuronal parameter space. PLoS Comput. Biol. 19:e1011212. doi: 10.1371/journal.pcbi.1011212, PMID: 37399220 PMC10353791

[ref49] Stanfield B. B. Caviness V. S. Cowan W. M. (1979). The organization of certain afferents to the hippocampus and dentate gyrus in normal and reeler mice. J. Comp. Neurol. 185, 461–483. doi: 10.1002/cne.901850304, PMID: 438367

[ref50] Steward O. (1989). Reorganization of neuronal connections following CNS trauma: principles and experimental paradigms. J. Neurotrauma 6, 99–152. doi: 10.1089/neu.1989.6.99, PMID: 2671393

[ref51] Steward O. Vinsant S. L. (1983). The process of reinnervation in the dentate gyrus of the adult rat: a quantitative electron microscopic analysis of terminal proliferation and reactive synaptogenesis. J. Comp. Neurol. 214, 370–386. doi: 10.1002/cne.902140403

[ref52] Takahashi N. Kitamura K. Matsuo N. Mayford M. Kano M. Matsuki N.. (2012). Locally synchronized synaptic inputs. Science 335, 353–356. doi: 10.1126/science.1210362 22267814

[ref53] Vivar C. van Praag H. (2013). Functional circuits of new neurons in the dentate gyrus. Front. Neural Circ. 7:15. doi: 10.3389/fncir.2013.00015, PMID: 23443839 PMC3580993

[ref54] Vlachos A. Bas Orth C. Schneider G. Deller T. (2012a). Time-lapse imaging of granule cells in mouse entorhino-hippocampal slice cultures reveals changes in spine stability after entorhinal denervation. J. Comp. Neurol. 520, 1891–1902. doi: 10.1002/cne.23017, PMID: 22134835

[ref55] Vlachos A. Becker D. Jedlicka P. Winkels R. Roeper J. Deller T. (2012b). Entorhinal denervation induces homeostatic synaptic scaling of excitatory postsynapses of dentate granule cells in mouse organotypic slice cultures. PLoS One 7:e32883. doi: 10.1371/journal.pone.0032883, PMID: 22403720 PMC3293910

[ref56] Vlachos A. Helias M. Becker D. Diesmann M. Deller T. (2013). NMDA-receptor inhibition increases spine stability of denervated mouse dentate granule cells and accelerates spine density recovery following entorhinal denervation in vitro. Neurobiol. Dis. 59, 267–276. doi: 10.1016/j.nbd.2013.07.018, PMID: 23932917

[ref57] Vuksic M. Del Turco D. Vlachos A. Schuldt G. Müller C. M. Schneider G.. (2011). Unilateral entorhinal denervation leads to long-lasting dendritic alterations of mouse hippocampal granule cells. Exp. Neurol. 230, 176–185. doi: 10.1016/j.expneurol.2011.04.011, PMID: 21536031

[ref58] Yap K. Drakew A. Smilovic D. Rietsche M. Paul M. H. Vuksic M.. (2020). The actin-modulating protein synaptopodin mediates long-term survival of dendritic spines. eLife 9:62944. doi: 10.7554/eLife.62944, PMID: 33275099 PMC7717903

[ref59] Zafirov S. Heimrich B. Frotscher M. (1994). Dendritic development of dentate granule cells in the absence of their specific extrinsic afferents. J. Comp. Neurol. 345, 472–480. doi: 10.1002/cne.903450312, PMID: 7929913

[ref60] Zhao C. Teng E. M. Summers R. G. Ming G. Gage F. H. (2006). Distinct morphological stages of dentate granule neuron maturation in the adult mouse hippocampus. J. Neurosci. 26, 3–11. doi: 10.1523/JNEUROSCI.3648-05.2006, PMID: 16399667 PMC6674324

[ref61] Zimmer J. Gähwiler B. H. (1984). Cellular and connective organization of slice cultures of the rat hippocampus and fascia dentata. J. Comp. Neurol. 228, 432–446. doi: 10.1002/cne.902280310, PMID: 6148364

[ref62] Ziv N. E. Smith S. J. (1996). Evidence for a role of dendritic filopodia in synaptogenesis and spine formation. Neuron 17, 91–102. doi: 10.1016/S0896-6273(00)80283-4, PMID: 8755481

