# Supplementary material for: Time-lapse imaging of identified granule cells in the mouse dentate gyrus after entorhinal lesion in vitro reveals heterogeneous cellular responses to denervation
Source: Front Neuroanat. 2025 Jan 21;18:1513511. doi: 10.3389/fnana.2024.1513511 (PMC11790675; doi:10.3389/fnana.2024.1513511)
Supplement: Supplementary file 1 [file Data_Sheet_1.pdf]

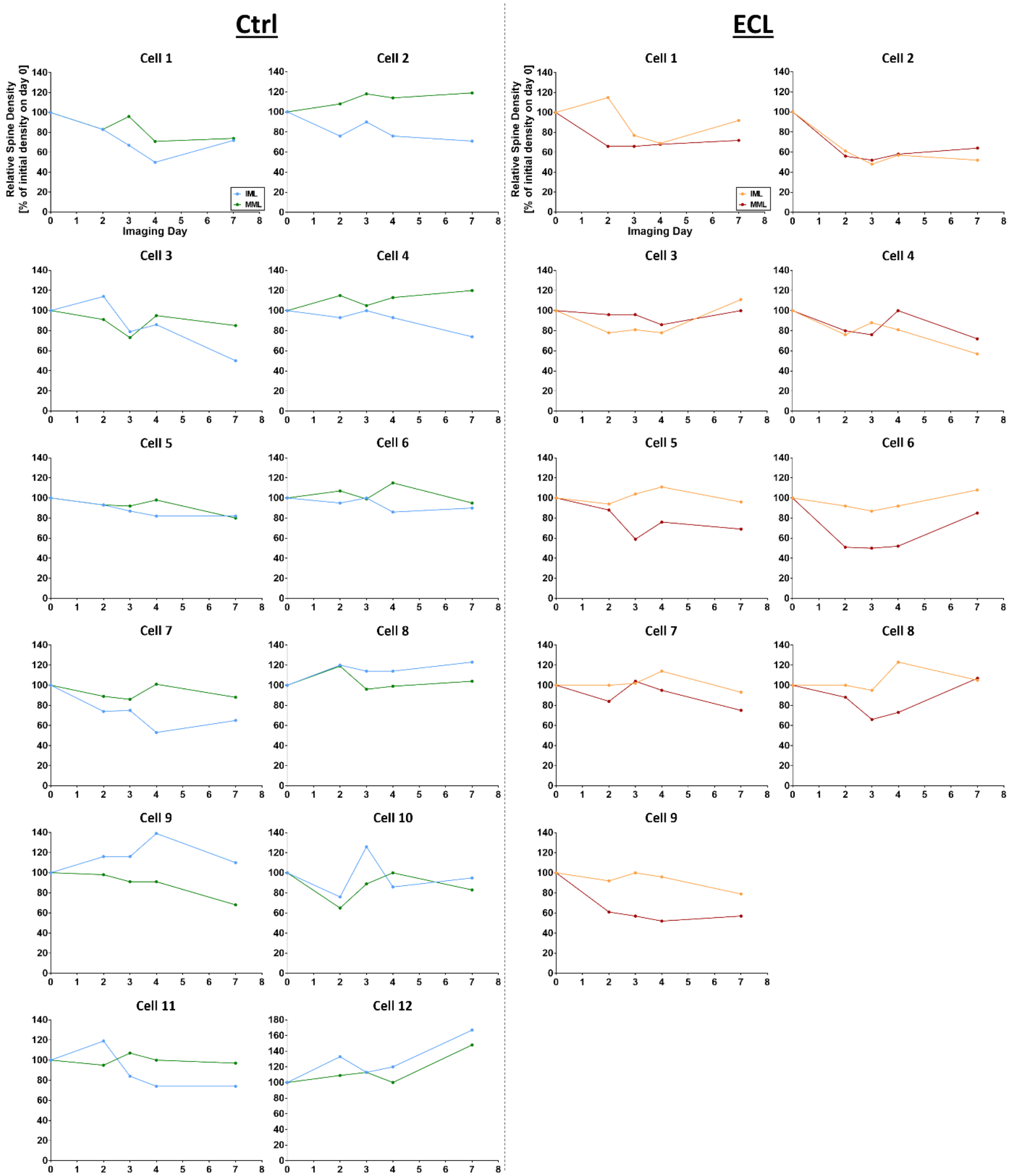

Supplement figure 1: Cell- and layer-specific spine density changes of single granule cells. Left: Spine density changes of single granule cells in non-denervated control (Ctrl) cultures. Dendritic segments in the IML are illustrated in blue and dendritic segments in the MML are illustrated in green. **Right:** Spine density changes of single granule cells in denervated (ECL) cultures. Spine density changes in the IML are illustrated in orange and spine density changes in the MML are illustrated in red.

Supplement Table 1: Spine density changes of dendritic segments relative to initial density on day 0

	**Control cultures [% of day 0]**												
	**Cell**	**1**	**2**	**3**	**4**	**5**	**6**	**7**	**8**	**9**	**10**	**11**	**12**
**IML**	Day 2	83	76	114	93	93	95	74	120	116	76	119	133
	Day 3	67	90	79	100	87	100	75	114	116	126	84	113
	Day 4	50	76	86	93	82	86	53	114	139	86	74	120
	Day 7	72	71	50	74	82	90	65	123	110	95	74	167
**MML**	Day 2	83	108	91	115	93	107	89	119	98	65	95	109
	Day 3	96	118	73	105	92	99	86	96	91	89	107	113
	Day 4	71	114	95	113	98	115	101	99	91	100	100	100
	Day 7	74	119	85	120	80	95	88	104	68	83	97	148
	**Denervated cultures [% of day 0]**												
	Cell	1	2	3	4	5	6	7	8	9			
**IML**	Day 2	115	61	78	76	94	92	100	100	92			
	Day 3	77	48	81	88	104	87	102	95	100			
	Day 4	69	57	78	81	111	92	114	123	96			
	Day 7	92	52	111	57	96	108	93	105	79			
**MML**	Day 2	66	56	96	80	88	51	84	88	61			
	Day 3	66	52	96	76	59	50	104	66	57			
	Day 4	68	58	86	100	76	52	95	73	52			
	Day 7	72	64	100	72	69	85	75	107	57			
